# Supplementary material for: Validation of the Malay version of the Diabetes Health Literacy Scale among Malaysian adults with type 2 diabetes mellitus
Source: PeerJ. 2025 Jul 28;13:e19660. doi: 10.7717/peerj.19660 (PMC12312687; doi:10.7717/peerj.19660)
Supplement: Supplemental Information 2 [file peerj-13-19660-s002.pdf]

Malay Version of Diabetes Health Literacy Scale **(DHLS-M)**

Arahan: Sila baca setiap kenyataan di bawah dan tandakan √ pada kotak yang paling sesuai untuk menunjukkan tahap persetujuan anda berkaitan kenyataan di bawah (Nota: tiada jawapan yang betul atau salah untuk semua kenyataan di bawah).

No	Kenyataan	Sangat Tidak setuju	Tidak setuju	Kurang setuju	Setuju	Sangat setuju
1	Saya boleh membaca dan memahami bahan pendidikan dan risalah tentang diabetes.					
2	Saya memahami maklumat bertulis tentang rawatan atau pemeriksaan diabetes yang disampaikan pada sesi janji temu.					
3	Saya memahami maklumat yang dicari berkenaan diabetes (contoh: daripada internet, surat khabar, majalah dan lain-lain).					
4	Saya memahami maklumat tentang pengurusan diabetes yang disampaikan oleh pengamal kesihatan (contoh: doktor, jururawat, ahli farmasi dan lain-lain).					
5	Saya boleh menilai sama ada sebarang maklumat berkenaan diabetes boleh dipercayai atau tidak.					
6	Saya mengetahui cara untuk mendapatkan preskripsi ubat saya dari hospital.					
7	Apabila terdapat perubahan pada jadual peribadi saya, saya tahu cara untuk mendapatkan tarikh atau masa janji temu yang baharu untuk pemeriksaan kesihatan.					
8	Saya tahu masa pengambilan ubat diabetes yang seterusnya.					
9	Saya boleh menentukan kandungan karbohidrat dalam setiap hidangan berpandukan label nutrisi pada bungkusan makanan.					
10	Saya boleh menilai paras gula darah saya sama ada berada dalam julat normal atau tidak.					
11	Saya boleh memahami maklumat tentang diabetes dalam bentuk kebarangkalian, nisbah atau graf.					
12	Apabila saya mempunyai soalan berkenaan diabetes, saya biasanya bertanya kepada pengamal kesihatan.					
13	Saya boleh menerangkan keadaan penyakit diabetes saya kepada pengamal kesihatan.					
14	Apabila makan di luar bersama rakan sekerja atau kawan, saya boleh menjelaskan sebab saya perlu mengamalkan diet diabetes.					
