# Supplementary material for: Validation of the Malay version of the Diabetes Health Literacy Scale among Malaysian adults with type 2 diabetes mellitus
Source: PeerJ. 2025 Jul 28;13:e19660. doi: 10.7717/peerj.19660 (PMC12312687; doi:10.7717/peerj.19660)
Supplement: Supplemental Information 3 [file peerj-13-19660-s003.pdf]

Diabetes Health Literacy Scale (English version) (Lee et al., 2018)

Please read each of the following items and mark √ in the space that most closely indicates how much you agree with it (note that there is no correct answer for any of the items).

No	Item	Not really	Slightly	Moderately	Quite a lot	Very much
1	I can read and understand the educational materials and booklets on diabetes.					
2	I understand the written information provided at an appointment for diabetes treatment or an examination.					
3	I comprehend the information I sought on diabetes.					
4	I understand the information on diabetes management provided by the health-care provider.					
5	I can judge if diabetes-related information is reliable.					
6	I can print out my prescription from an automated prescription machine at the hospital.					
7	When a change occurs in my personal schedule, I can alter the appointment date or time for a medical checkup.					
8	I can calculate the next time to take diabetes medication.					
9	I can determine the carbohydrate content per serving from the nutrition label on food packaging.					
10	I can interpret if my blood-glucose level is within the normal range.					
11	I can understand information on diabetes presented as probabilities, ratios, or on graphs.					
12	When I have a question about diabetes, I usually ask a health-care provider.					
13	I can explain my diabetes condition to a health-care provider.					
14	When eating out with colleagues or friends, I can convey the reason why I should have a diabetic diet.					
