# Supplementary material for: Validation of the Malay version of the Diabetes Health Literacy Scale among Malaysian adults with type 2 diabetes mellitus
Source: PeerJ. 2025 Jul 28;13:e19660. doi: 10.7717/peerj.19660 (PMC12312687; doi:10.7717/peerj.19660)
Supplement: Supplemental Information 4 [file peerj-13-19660-s004.docx]

STROBE Statement—checklist of items that should be included in reports of observational studies

|  | Item No. | Recommendation | Page  No. | Relevant text from manuscript |
| --- | --- | --- | --- | --- |
| **Title and abstract** | 1 | (*a*) Indicate the study’s design with a commonly used term in the title or the abstract | 1 | A cross-sectional study |
|  |  | (*b*) Provide in the abstract an informative and balanced summary of what was done and what was found | 1 – 2 | The Diabetes Health Literacy Scale (DHLS) was translated and culturally adapted into Malay, followed by a cross-sectional study which was conducted using a self-administered questionnaire among the adults with T2DM in Hospital Universiti Sains Malaysia (USM). Confirmatory factor analysis (CFA) and correlation analysis were performed. DHLS-M which consisted of three subscales and 13 items, is a valid and reliable instrument. |
| Introduction | | | |  |
| Background/rationale | 2 | Explain the scientific background and rationale for the investigation being reported | 2 | There is no validated diabetes-specific health literacy instrument in Malay. An instrument measuring health literacy in specific context such as diabetes is important because different contexts may require different abilities and skills. The Diabetes Health Literacy Scale (DHLS) is a diabetes-specific health literacy instrument developed by Lee et al. (2018) for measuring diabetes health literacy among adults with T2DM. |
| Objectives | 3 | State specific objectives, including any prespecified hypotheses | 3 | The objectives of this study were to translate and adapt the DHLS into the Malay language, determine the construct validity and reliability of the Malay version of the DHLS (DHLS-M) among Malaysian adults with T2DM, and examine the convergent validity by correlating the DHLS-M with the Malay version of the Michigan Diabetes Knowledge Test (MDKT) and the Malay version of the short form European Health Literacy Survey Questionnaire (HLS-SF12). The researchers hypothesized that the DHLS-M is valid and reliable in measuring diabetes health literacy among Malaysian adults with T2DM and there is a significant correlation between the score of the Malay version of the MDKT, the Malay version of the HLS-SF12 and the DHLS-M. |
| Methods | | | |  |
| Study design | 4 | Present key elements of study design early in the paper | 3 | A cross-sectional study |
| Setting | 5 | Describe the setting, locations, and relevant dates, including periods of recruitment, exposure, follow-up, and data collection | 3 | A cross-sectional study was conducted among adults with T2DM receiving treatment at the Family Medicine Clinic and Diabetes Mellitus Specialist Clinic, Hospital Universiti Sains Malaysia (USM) from January to March 2024. |
| Participants | 6 | (*a*) *Cohort study*—Give the eligibility criteria, and the sources and methods of selection of participants. Describe methods of follow-up  *Case-control study*—Give the eligibility criteria, and the sources and methods of case ascertainment and control selection. Give the rationale for the choice of cases and controls  *Cross-sectional study*—Give the eligibility criteria, and the sources and methods of selection of participants | 3 | The inclusion criteria were Malaysian of age more than 18 years old, diagnosed with T2DM for at least six months and able to read, understand and response to the questionnaire which was in Malay. The exclusion criteria were those with type 1 diabetes mellitus and gestational diabetes. A total of 278 participants were recruited using convenience sampling in this study. |
|  |  | (*b*) *Cohort study*—For matched studies, give matching criteria and number of exposed and unexposed  *Case-control study*—For matched studies, give matching criteria and the number of controls per case | Not applicable | Not applicable |
| Variables | 7 | Clearly define all outcomes, exposures, predictors, potential confounders, and effect modifiers. Give diagnostic criteria, if applicable | 3 – 4 | Socio-demographic and diabetes information; diabetes health literacy; health literacy; diabetes knowledge |
| Data sources/ measurement | 8* | For each variable of interest, give sources of data and details of methods of assessment (measurement). Describe comparability of assessment methods if there is more than one group | 3 – 5 | Socio-demographic and diabetes information; Diabetes Health Literacy Scale (In this study, the English version of DHLS was translated into Malay); Malay version of short form European Health Literacy Survey Questionnaire; Malay version of Michigan Diabetes Knowledge Test. Data collection was performed using a self-administered questionnaire. The participants’ HbA1c level was obtained from their laboratory records. |
| Bias | 9 | Describe any efforts to address potential sources of bias | 5 | A copy of the participant information sheet, informed consent form (ICF) and questionnaire was given to them. They were briefed about the study and informed to fill up the ICF and the questionnaire if they agreed to participate. The return of the signed ICF and the questionnaire was considered complete participation. |
| Study size | 10 | Explain how the study size was arrived at | 3 | The sample size of this study was calculated using confirmatory factor analysis (CFA) by root mean square error of approximation (RMSEA) in the Sample Size Calculator (web) by Arifin (2023), which was based on Kim’s (2005) method. By using expected RMSEA = 0.05, number of items = 14, number of factors = 3, significant level (α) = 0.05, power (1 – β) = 80%, expected dropout rate = 30%, the estimated sample size was 278. |
| Quantitative variables | 11 | Explain how quantitative variables were handled in the analyses. If applicable, describe which groupings were chosen and why | Table 1 | HbA1c level: Controlled, HbA1c ˂ 7.0%; Uncontrolled, HbA1c ≥ 7.0% |
| Statistical methods | 12 | (*a*) Describe all statistical methods, including those used to control for confounding | 5 – 6 | Descriptive statistics; Confirmatory factor analysis (CFA); Spearman’s correlation |
|  |  | (*b*) Describe any methods used to examine subgroups and interactions | Not applicable | Not applicable |
|  |  | (*c*) Explain how missing data were addressed | 6 | A total of 250 participants with complete data on DHLS-M were included in the analysis. |
|  |  | (*d*) *Cohort study*—If applicable, explain how loss to follow-up was addressed  *Case-control study*—If applicable, explain how matching of cases and controls was addressed  *Cross-sectional study*—If applicable, describe analytical methods taking account of sampling strategy | Not applicable | Not applicable |
|  |  | (*e*) Describe any sensitivity analyses | Not applicable | Not applicable |
| **Results** |  |  |  |  |
| Participants | 13* | (a) Report numbers of individuals at each stage of study—eg numbers potentially eligible, examined for eligibility, confirmed eligible, included in the study, completing follow-up, and analysed | 3 & 6 | A total of 278 participants were recruited. A total of 250 participants with complete data on DHLS-M were included in the analysis. |
|  |  | (b) Give reasons for non-participation at each stage | Not applicable | Not applicable |
|  |  | (c) Consider use of a flow diagram | Not applicable | Not applicable |
| Descriptive data | 14* | (a) Give characteristics of study participants (eg demographic, clinical, social) and information on exposures and potential confounders | Table 1 | Table 1 |
|  |  | (b) Indicate number of participants with missing data for each variable of interest | Table 1 | Table 1 |
|  |  | (c) *Cohort study*—Summarise follow-up time (eg, average and total amount) | Not applicable | Not applicable |
| Outcome data | 15* | *Cohort study*—Report numbers of outcome events or summary measures over time | Not applicable | Not applicable |
|  |  | *Case-control study—*Report numbers in each exposure category, or summary measures of exposure |  |  |
|  |  | *Cross-sectional study—*Report numbers of outcome events or summary measures |  |  |
| Main results | 16 | (*a*) Give unadjusted estimates and, if applicable, confounder-adjusted estimates and their precision (eg, 95% confidence interval). Make clear which confounders were adjusted for and why they were included | Table 2 & Table 5 | Table 2 & Table 5 |
|  |  | (*b*) Report category boundaries when continuous variables were categorized | Not applicable | Not applicable |
|  |  | (*c*) If relevant, consider translating estimates of relative risk into absolute risk for a meaningful time period | Not applicable | Not applicable |
| Other analyses | 17 | Report other analyses done—eg analyses of subgroups and interactions, and sensitivity analyses | Not applicable | Not applicable |
| **Discussion** |  |  |  |  |
| Key results | 18 | Summarise key results with reference to study objectives | 7 – 8 | CFA was performed to confirm the construct validity and reliability of the DHLS-M. The model fit of the initial model was insufficient. A problematic item, item B1 was identified and removed. The model fit of the revised model was improved with three fit indices (χ2/df, CFI, TLI) within the acceptable threshold value, and accepted as the final model. The factor loading of all the items were more than 0.4 and statistically significant. The correlation between each pair of subscales were less than 0.85 except the correlation between informational health literacy and communicative health literacy subscale. The composite reliability of the three subscales were more than 0.7, showed good reliability. |
| Limitations | 19 | Discuss limitations of the study, taking into account sources of potential bias or imprecision. Discuss both direction and magnitude of any potential bias | 9 | This study was conducted using a self-administered questionnaire which consisted of two self-reported instruments and a performance-based instrument. For self-reported instruments, participants tend to provide social desired or positive responses which result in overestimation of health literacy. some of the participants required assistance to read the questionnaire aloud and sought clarification during the administration of the questionnaire. This may introduce bias to the study. |
| Interpretation | 20 | Give a cautious overall interpretation of results considering objectives, limitations, multiplicity of analyses, results from similar studies, and other relevant evidence | 9 | The final model of DHLS-M showed an acceptable model fit, valid and reliable. |
| Generalisability | 21 | Discuss the generalisability (external validity) of the study results | 9 | The data were collected from a single hospital. More than 90% of the participants were Malay. The non-Malay participants in this study were less than 10%. Thus, the results cannot be generalised to the whole diabetes population in Malaysia. |
| **Other information** | |  |  |  |
| Funding | 22 | Give the source of funding and the role of the funders for the present study and, if applicable, for the original study on which the present article is based | Not applicable | Not applicable |

*Give information separately for cases and controls in case-control studies and, if applicable, for exposed and unexposed groups in cohort and cross-sectional studies.

**Note:** An Explanation and Elaboration article discusses each checklist item and gives methodological background and published examples of transparent reporting. The STROBE checklist is best used in conjunction with this article (freely available on the Web sites of PLoS Medicine at http://www.plosmedicine.org/, Annals of Internal Medicine at http://www.annals.org/, and Epidemiology at http://www.epidem.com/). Information on the STROBE Initiative is available at www.strobe-statement.org.
